# Outdoor physical activity, compliance with the physical activity, screen time, and sleep duration recommendations, and excess weight among adolescents

**DOI:** 10.1002/osp4.389

**Published:** 2019-12-04

**Authors:** Hugues Sampasa‐Kanyinga, Ian Colman, Hayley A. Hamilton, Jean‐Philippe Chaput

**Affiliations:** ^1^ School of Epidemiology and Public Health University of Ottawa Ottawa Ontario Canada; ^2^ Healthy Active Living and Obesity Research Group Children's Hospital of Eastern Ontario Research Institute Ottawa Ontario Canada; ^3^ Institute for Mental Health Policy Research Centre for Addiction and Mental Health Toronto Ontario Canada; ^4^ Dalla Lana School of Public Health University of Toronto Toronto Ontario Canada

**Keywords:** exercise, movement behaviours, obesity, outdoor time

## Abstract

**Background:**

Spending time outdoor has been identified as an important way to achieve the physical activity required for maintaining and improving health and to lower sedentary time among young children. However, evidence of such relationships in adolescents is particularly limited. This study investigated the relationships between frequency of outdoor physical activity after school, compliance with the physical activity, screen time, and sleep duration recommendations, and overweight/obesity among adolescents.

**Methods:**

A total of 10 028 middle and high school students (mean age of 15.2 y) self‐reported the number of weekdays they spent physically active outdoors after school. Physical activity, screen time, sleep duration, height, and weight were self‐reported. Logistic regression models for the total sample and stratified by sex were adjusted for important covariates**.**

**Results:**

Overall, there was a positive gradient between the number of weekdays spent physically active outdoor after school and compliance with the physical activity (more than or equal to 60 min/day at moderate‐to‐vigorous intensity) and screen time (less than or equal to 2 h/day) recommendations while a negative gradient with overweight/obesity was observed. Significant sex differences were observed in the associations of outdoor physical activity after school with adherence to the sleep duration and physical activity recommendations. For example, outdoor physical activity after school on all 5 days was associated with greater odds of compliance with the sleep duration recommendation among males (OR = 1.53; 95% CI, 1.01‐2.31), but not females (OR = 0.92; 95% CI, 0.65‐1.30).

**Conclusions:**

Results suggest that outdoor physical activity after school could be a behavioural target to increase compliance with the physical activity and screen time recommendations and to possibly tackle excess weight among adolescents.

## 1 INTRODUCTION

Physical activity is essential for healthy development of children and adolescents and is associated with important physical, mental, and social health benefits.^1^, ^2^ However, more than 80% of the world's adolescent population is insufficiently physically active.^3^ A recent study suggests that the majority (76%) of Canadian youth aged 12 to 17 years do not meet the current physical activity guidelines of at least 60 minutes of moderate‐to‐vigorous physical activity (MPVA) per day compared with 52% of 5 to 11 year‐olds.^4^ Today's children and adolescents spend a large amount of their time sedentary, mainly in the form of screen time behaviours such as smartphone, tablet, and recreational computer use, TV viewing, and video gaming. This is alarming because physical activity and sedentary behaviour habits formed during childhood may extend into adulthood.^5^, ^6^ Spending time outdoor has been identified as an important way to achieve the physical activity required for maintaining and improving health and to lower sedentary time among young children.^7^, ^8^, ^9^, ^10^ For example, a study of 1159 children aged 7 to 14 years found that children reporting more time outdoors were more active, less sedentary, and less likely to have peer relationship problems compared with those who spent less time outdoors.^8^ However, evidence of such relationships in adolescents is particularly limited.

Surprisingly, previous research has focused on examining the influence of outdoor time on physical activity, screen time, and other physical and social outcomes^7^, ^8^; however, its relationship with sleep duration has received very little attention. Sleep is an essential component of healthy development and is required for physical and mental health.^11^ Short sleep duration has become common in modern societies, with recent research indicating that nearly one third of Canadian children and adolescents sleep less than recommended by public health authorities for optimal health.^12^, ^13^ A study using a sample of 433 Canadian children aged 10 to 13 years found that outdoor active play was a modest predictor of time in bed but it was not associated with sleep duration, sleep chronology, or sleep efficiency.^14^ Physical activity has been indicated to contribute to better sleep throughout the lifespan.^15^ Thus, frequent outdoor physical activity could be associated with better adherence to the sleep duration recommendation in adolescents.

Childhood obesity is a public health concern that has long‐term implications for physical and mental health.^16^ To date, few studies have investigated whether outdoor physical activity is directly associated with body weight status among adolescents. Most studies focused on preschool and young children and found that more time playing outdoors was associated with lower body weight compared with those who spend less time.^17^, ^18^, ^19^, ^20^ However, research has shown that children spend more outdoor time than adolescents, and thus cumulate more physical activity time.^21^, ^22^ A study of over 1600 Indian school children aged 10 to 15 years found that the prevalence of both overweight and obesity were greater among students who did not participate in outdoor sports.^23^ However, this study did not include older adolescents and may thus have overestimated the strength of this association.

Moreover, previous studies have mainly focused on time spent outdoors. This can conflate physical activity with many other activities that adolescents engage in while outside.^22^ A more precise measure that includes the specific type of activity youth engage in while outside, such as “outdoor physical activity,” and the frequency of such an activity at the population level of adolescents is needed. This is important because technological and societal changes have resulted in secular trends that have impacted the types of activities performed by children.^24^ There is a decline in time spent playing outdoors,^25^ a switch from unstructured outdoor to structured indoor activities (eg, competitive sports and music practice)^7^ and a substantial increase in time spent using electronic media in recent decades.^24^

Thus, the present study investigated the relationships between frequency of outdoor physical activity after school, adherence to the physical activity, screen time, and sleep duration recommendations, and overweight/obesity among a representative sample of adolescents. We hypothesized that more frequent outdoor physical activity after school would result in better adherence to the physical activity, screen time, and sleep duration recommendations and lower overweight/obesity prevalence.

## 2 METHODS

Data for this study were obtained from the 2017 Ontario Student Drug Use and Health Survey (OSDUHS), a school‐based study of students in grades 7 through 12 (aged 11‐20 y or older) in publicly funded schools across Ontario, Canada. Conducted every 2 years since 1977, the OSDUHS is the longest ongoing school survey of adolescents in Canada and one of the longest in the world. The OSDUHS uses a two‐stage (school, class) stratified (region and school type) cluster design and sampled 11 435 students from 52 school boards, 214 schools, and 764 classrooms in 2017. The student participation rate was 63%, which is above average for a student survey that required active consent from a parent or guardian.^26^ Reasons for nonparticipation included absenteeism (11%) and parental refusal or unreturned consent forms (26%).^27^ Detailed information on the study design and methods are available elsewhere.^27^ All participants provided their signed assent in addition to parentally signed consent for those aged under 18. The survey received research ethics approval from the Centre for Addiction and Mental Health and from York University, as well as existing research review committees of participating school boards.

### 2.1 Measures

#### 2.1.1 Outdoor physical activity

Frequency of physical activity outdoor after school was measured using an item that asked how many of the last five school days students were physically active outside after school, such as playing games or sports (does not include travel mode home from school or any inside activities). Response options included 0, 1 to 2, 3 to 4, and all 5 days.

#### 2.1.2 Physical activity recommendation

Physical activity was assessed using a question that asked participants how many of the last 7 days they were physically active for at least 60 minutes each day. Students were categorized as meeting the current physical activity recommendation of at least 60 minutes per day of MVPA on all 7 days, or not.^28^

#### 2.1.3 Screen time recommendation

Students were asked how many hours a day, on average, in their free time they spent watching TV/movies/videos, playing video/computer games, texting, emailing, or surfing the Internet in the last 7 days. Students meeting the screen time guideline of less than or equal to 2 hours per day were compared with those not meeting the screen time guideline.^28^

#### 2.1.4 Sleep duration recommendation

Sleep duration was assessed using a question that asked participants how many hours of sleep on an average school night they usually get. A binary variable was created to contrast students who had a sleep duration that met or exceeded the recommended range (9‐11 h/night for 11‐ to 13‐year‐olds; 8‐10 h/night for 14‐ to17‐year‐olds; or 7‐9 h/night for those 18 years of age or older) with those who reported a short sleep duration (less than 9 h/night for 11‐ to 13‐year‐olds; less than 8 h/night for 14‐ to 17‐year‐olds; or less than 7 h/night for those 18 years of age or older).^28^, ^29^

#### 2.1.5 Body mass index

Students self‐reported their height and weight and the response options were precoded. The item that measured height provided a total of 27 options in feet/centimetres, while the one that measured weight provided 42 options in 5‐lb (or equivalent kilogramme) intervals. We used the midpoint of the height and the weight response categories and calculated body mass index (BMI) as weight divided by the square of height (kilogrammes/metres^2^). Overweight and obesity were defined using the World Health Organization's sex‐ and age‐specific BMI cut points established for children and youth^30^ and the International Classification for students over 19 years (n = 12). A dichotomous variable was created contrasting “overweight or obesity” with “normal weight,” which included for the present study both the normal and underweight categories. A sensitivity analysis excluding the underweight students (n = 506) provided similar results.

### 2.2 Covariates

Covariates included age (in years), sex (male/female), ethnicity (white, black, East/Southeast Asian, South Asian, and others), subjective socioeconomic status (SES), parental education and support, and perceived school economic status. Subjective SES was assessed using the youth version of the MacArthur Scale of Subjective Social Status.^31^ Participants indicated the rung that best represents their family's place in Canadian society with respect to money, education, and occupation. Responses were treated as a scale ranging from 1 to 10; the higher the rung, the higher the perceived family subjective social status, including more money, higher education, and highly respected occupations. Educational level of the father and mother were measured by the following items: “How far did your father go in school?” and “How far did your mother go in school?” Response options were treated as a scale ranging from 8 years (did not attend high school) to 16 years (graduated university) for each parent. Means for father (14.3 y; SD = 2.0) and mother (14.5 y; SD = 1.8) education levels were used when respective level of education was not available (father's: 22.7% and mother's: 18%). Parental support was measured by an item that asked students to determine how often they talked about their problems or feelings with at least one of their parents. Response options ranged from “Always” to “Never”. Responses were reverse coded, so that higher scores indicate higher levels of parental support. School subjective social status was measured using the MacArthur Scale of Subjective Social Status.^31^, ^32^ The questionnaire included a 10‐rung ladder to represent the social hierarchy at school. At the top of the ladder are the people in school with the most respect and the highest standing, and at the bottom of the ladder are the people who no one respects and no one wants to hang out with. Response options were treated as a scale ranging from 1 to 10. Covariates were selected based on previous publications,^8^, ^22^ their availability in the dataset, and their association with the dependent and independent variables.

### 2.3 Data analysis

Taylor series methods within Stata 14.0 (Stata Corporation, College Station, Texas) were utilized for analyses to account for the complex survey sample design and obtain unbiased variances and point estimates. Analyses included population weights to adjust for the unequal probability of selection. Conventional descriptive statistics, such as proportions and means, were used to describe the sample. Pearson χ^2^ test, adjusted for survey design and transformed into an *F* statistic, examined sex differences in categorical data compared with the adjusted Wald test for continuous data. Separate two‐way interactions were used to test interactions involving outdoor physical activity and sex for each of the outcome variables. Given those of physical activity and sleep duration were statistically significant, subsequent analyses were stratified by sex. Univariate and multivariate logistic regression were used to examine the associations between the number of days spent physically active outdoors after school with the outcome variables of compliance with the physical activity, screen time, and sleep duration recommendations, and overweight/obesity among adolescents. Potential confounders included age, sex, ethnic background, subjective socioeconomic status, father education level, mother education level, parental support, and school subjective social status. Odds ratios (OR), 95% confidence intervals (CI), and *P* values are reported. Separate two‐way interactions were used to test interactions involving outdoor physical activity variables and sex for each of the outcome variables. Only participants with complete information on all variables were included in the current analyses. Missing values (n = 1407, representing 12.3% of the total sample) represented less than 5% for any of the variables examined, and the probability of the outcome variables were not significantly different between missing and nonmissing cases.

## 3 RESULTS

Descriptive characteristics of the sample stratified by sex are outlined in Table 1. About half of the sample was female (49%), over 54.6% identified themselves as white, and the average age was 15 years. One‐in‐five students (20.9%) reported being physically active outdoor in all 5 days. The majority of students were in high school (75.5%) and did not meet the physical activity (76.7%), screen time (66.6%), or sleep duration (66.3%) recommendations. Nearly one third of the respondents (30.7%) were classified as overweight/obese. Females were more likely to perceive higher levels of parental support and to report a higher school subjective social status than their male counterparts, while males were more likely than females to be physically active outdoor after school on all 5 days of the week and to meet physical activity and sleep duration recommendations.

**Table 1:** Descriptive characteristics of the sample

	Total sample	Males	Females	*P* value^a^
	n (%)	n (%)	n (%)	
Total	10028 (100)	4333 (51.3)	5695 (48.8)	
Age				
Mean (SD)	15.2 (1.8)	15.2 (1.7)	15.1 (1.9)	.221
Grade				
7	1418 (11.7)	614 (11.0)	804 (12.4)	.819
8	1702 (12.7)	801 (13.2)	901 (12.1)	
9	1927 (16.2)	784 (15.9)	1143 (16.6)	
10	1758 (17.3)	794 (17.5)	964 (17.0)	
11	1592 (18.2)	665 (17.6)	927 (18.8)	
12	1631 (24.0)	675 (24.9)	956 (23.2)	
Ethnic background				
White	5813 (56.4)	2623 (58.7)	3190 (53.9)	.197
Black	759 (9.7)	295 (9.4)	464 (10.1)	
East/South‐East Asian	951 (8.8)	376 (8.4)	575 (9.2)	
South Asian	806 (7.0)	336 (7.1)	470 (6.9)	
Other	1699 (18.1)	703 (16.3)	996 (19.9)	
Subjective socioeconomic status				
Mean (SD)	6.9 (1.7)	7.0 (1.6)	6.9 (1.8)	.571
Father education level				
Mean (SD)	13.0 (3.0)	13.1 (2.8)	12.9 (3.3)	.208
Mother education level				
Mean (SD)	13.6 (2.9)	13.5 (2.7)	13.6 (3.0)	.474
Parental support				
Mean (SD)	2.9 (1.2)	2.8 (1.1)	3.1 (1.3)	<.001
School subjective social status				
Mean (SD)	7.1 (1.9)	7.2 (1.7)	7.9 (2.0)	<.001
Frequency of physical activity outdoor				
0 d	2453 (25.3)	782 (18.2)	1671 (32.7)	<.001
1 to 2 ds	3074 (30.5)	1177 (29.2)	1897 (31.9)	
3 to 4 d	2458 (23.4)	1148 (25.0)	1310 (21.6)	
All 5 d	2043 (20.9)	1226 (27.6)	817 (13.7)	
Meet the physical activity recommendation				
Do not meet	7685 (76.7)	2968 (69.6)	4717 (84.3)	<.001
Meet	2343 (23.3)	1365 (30.4)	978 (15.7)	
Meet the screen time recommendation				
Do not meet	6547 (66.6)	2763 (66.0)	3784 (67.2)	.323
Meet	3481 (33.5)	1570 (34.1)	1911 (32.8)	
Meet the sleep duration recommendation				
Do not meet	6652 (66.3)	2680 (62.5)	3972 (70.4)	<.001
Meet	3376 (33.7)	1653 (37.5)	1723 (29.7)	
BMI categories				
Normal weight	7114 (69.3)	2920 (67.0)	4194 (71.7)	.056
Overweight/obese	2914 (30.7)	1413 (33.0)	1501 (28.3)	

*Note*. Data are shown as column %, unless otherwise indicated.

Abbreviations: BMI, body mass index; SD, standard deviation.

*P* values refer to differences between males and females. A Pearson χ^2^ test, adjusted for survey design and transformed into *F*‐statistic, examined sex differences in categorical data compared with an adjusted Wald test for continuous data.

Results from univariate and multivariate logistic regression analyses that examined the associations between the number of days spent physically active outdoors after school with the outcome variables of compliance with the physical activity, screen time, and sleep duration recommendations, and overweight/obesity are outlined in Tables 2, 3, 4, 5. Overall, before and after adjusting for covariates, the number of days spent physically active outdoors showed a positive gradient with compliance with the physical activity (Table 2) and screen time (Table 3) recommendations and a negative gradient with overweight/obesity (Table 5). Significant sex differences were observed in the associations of outdoor physical activity after school with adherence to the sleep duration (Table 4) and physical activity (Table 1) recommendations.

**Table 2:** Frequency of physical activity outdoor after school and compliance with the physical activity recommendation among adolescents

	Total N = 10 028	Males N = 4333	Females N = 5695
	OR (95% CI)	OR (95% CI)	OR (95% CI)
Unadjusted			
Frequency of physical activity outdoor			
0 day	1 (reference)	1 (reference)	1 (reference)
1 to 2 days	2.26 (1.71‐2.98)***	1.61 (1.14‐2.27)**	2.93 (1.94‐4.44)***
3 to 4 days	6.02 (4.25‐8.55)***	5.09 (3.10‐8.36)***	6.05 (4.08‐8.99)***
All 5 days	42.08 (31.06‐57.01)***	35.45 (24.79‐50.68)***	38.03 (25.7‐56.18)***
Adjusted			
Frequency of physical activity outdoor			
0 day	1 (reference)	1 (reference)	1 (reference)
1 to 2 days	1.90 (1.43‐2.51)***	1.50 (1.05‐2.16)*	2.50 (1.66‐3.77)***
3 to 4 days	4.69 (3.32‐6.63)***	4.59 (2.68‐7.87)***	4.70 (3.14‐7.04)***
All 5 days	31.85 (23.40‐43.35)***	31.82 (21.39‐47.34)***	31.48 (20.86‐47.51)***
Age	0.85 (0.80‐0.89)***	0.86 (0.79‐0.92)***	0.83 (0.77‐0.90)***
Sex			
Female	1 (reference)		
Male	1.60 (1.32‐1.94)***		
Ethnic background			
White	1 (reference)	1 (reference)	1 (reference)
Black	0.82 (0.57‐1.17)	0.90 (0.54‐1.50)	0.69 (0.40‐1.78)
East/South‐East Asian	0.81 (0.63‐1.04)	0.67 (0.42‐1.08)	1.02 (0.64‐1.62)
South Asian	0.87 (0.62‐1.21)	0.78 (0.53‐1.16)	1.00 (0.66‐1.54)
Other	0.67 (0.53‐0.84)**	0.59 (0.42‐0.82)**	0.81 (0.55‐1.18)
Subjective socioeconomic status	1.03 (0.95‐1.12)	1.02 (0.92‐1.13)	1.05 (0.95‐1.18)
Father education level	1.04 (0.99‐1.09)	1.04 (0.98‐1.10)	1.04 (0.97‐1.11)
Mother education level	0.99 (0.94‐1.38)	0.98 (0.91‐1.05)	1.01 (0.95‐1.08)
Parental support	0.90 (0.82‐0.99)*	0.88 (0.77‐1.01)	0.92 (0.82‐1.04)
School subjective social status	1.05 (0.99‐1.11)	1.05 (0.98‐1.12)	1.04 (0.94‐1.16)

* *P* < .05.

** *P* < .01.

*** *P* < .001.

**Table 3:** Frequency of physical activity outdoor after school and compliance with the screen time recommendation among adolescents

	Total N = 10028	Males N = 4333	Females N = 5695
	OR (95% CI)	OR (95% CI)	OR (95% CI)
Unadjusted			
Frequency of physical activity outdoor			
0 day	1 (reference)	1 (reference)	1 (reference)
1 to 2 days	1.33 (0.98‐1.81)	1.31 (0.92‐1.86)	1.41 (0.99‐2.03)
3 to 4 days	2.04 (1.51‐2.78)***	2.17 (1.55‐3.03)***	2.07 (1.37‐3.14)**
All 5 days	3.59 (2.65‐4.88)***	4.16 (2.61‐6.61)***	3.23 (2.46‐4.23)***
Adjusted			
Frequency of physical activity outdoor			
0 day	1 (reference)	1 (reference)	1 (reference)
1 to 2 days	1.18 (0.93‐1.51)	1.14 (0.84‐1.56)	1.28 (0.97‐1.68)
3 to 4 days	1.69 (1.34‐2.14)***	1.79 (1.32‐2.41)***	1.73 (1.26‐2.38)**
ll 5 days	3.02 (2.26‐4.04)***	3.35 (2.10‐5.36)***	2.76 (2.14‐3.54)***
Age	0.95 (0.90‐0.99)*	0.92 (0.87‐0.97)**	0.98 (0.91‐1.05)
Sex			
Female	1 (reference)		
Male	0.89 (0.81‐0.98)*		
Ethnic background			
White	1 (reference)	1 (reference)	1 (reference)
Black	0.82 (0.48‐1.40)	0.87 (0.38‐1.97)	0.78 (0.51‐1.18)
East/South‐East Asian	0.61 (0.47‐0.80)***	0.59 (0.39‐0.91)*	0.64 (0.48‐0.86)**
South Asian	0.93 (0.76‐1.13)	0.83 (0.59‐1.15)	1.05 (0.83‐1.34)
Other	0.88 (0.70‐1.11)	0.67 (0.51‐0.86)**	1.11 (0.74‐1.68)
Subjective socioeconomic status	1.11 (1.06‐1.16)***	1.11 (1.03‐1.20)**	1.10 (1.03‐1.18)**
Father education level	1.02 (0.99‐1.06)	1.03 (0.98‐1.08)	1.02 (0.95‐1.08)
Mother education level	1.00 (0.95‐1.05)	1.00 (0.95‐1.06)	1.00 (0.94‐1.06)
Parental support	1.10 (1.02‐1.19)*	1.09 (1.00‐1.18)*	1.11 (0.98‐1.27)
School subjective social status	1.05 (0.99‐1.10)	1.01 (0.95‐1.07)	1.08 (1.00‐1.17)*

* *P* < .05.

** *P* < .01.

*** *P* < .001.

**Table 4:** Frequency of physical activity outdoor after school and compliance with the sleep duration recommendation among adolescents

	Total N = 10028	Males N = 4333	Females N = 5695
	OR (95% CI)	OR (95% CI)	OR (95% CI)
Unadjusted			
Frequency of physical activity outdoor			
0 day	1 (reference)	1 (reference)	1 (reference)
1 to 2 days	1.47 (1.17‐1.85)**	1.51 (1.06‐2.15)*	1.38 (1.10‐1.71)**
3 to 4 days	1.45 (1.14‐1.85)**	1.45 (1.01‐2.08)*	1.36 (0.98‐1.88)
All 5 days	1.72 (1.34‐2.19)***	1.84 (1.31‐2.58)***	1.23 (0.91‐1.68)
Adjusted			
Frequency of physical activity outdoor			
0 day	1 (reference)	1 (reference)	1 (reference)
1 to 2 days	1.24 (0.99‐1.55)	1.38 (0.95‐2.01)	1.11 (0.87‐1.42)
3 to 4 days	1.12 (0.87‐1.45)	1.25 (0.81‐1.92)	1.00 (0.70‐1.45)
All 5 days	1.24 (0.95‐1.61)	1.53 (1.01‐2.31)*	0.92 (0.65‐1.30)
Age	0.96 (0.92‐0.99)*	098 (0.91‐1.05)	0.92 (0.86‐0.99)*
Sex			
Female	1 (reference)		
Male	1.46 (1.28‐1.66)***		
Ethnic background			
White	1 (reference)	1 (reference)	1 (reference)
Black	0.84 (0.62‐1.23)	1.25 (0.91‐1.05)	0.49 (0.36‐0.65)***
East/Southeast Asian	0.82 (0.66‐1.00)	0.73 (0.81‐0.97)*	0.91 (0.67‐1.23)
South Asian	0.83 (0.66‐1.04)	0.90 (0.63‐1.28)	0.73 (0.53‐1.02)
Other	0.89 (0.74‐1.07)	0.85 (0.61‐1.19)	0.88 (0.70‐1.11)
Subjective socioeconomic status	1.14 (1.06‐1.22)***	1.15 (1.07‐1.23)***	1.13 (1.04‐1.22)**
Father education level	0.99 (0.97‐1.02)	1.03 (1.00‐1.07)	0.95 (0.92‐0.98)
Mother education level	0.98 (0.96‐1.01)	0.97 (0.92‐1.02)	0.99 (0.95‐1.03)
Parental support	1.20 (1.13‐1.26)***	1.16 (1.07‐1.25)***	1.26 (1.17‐1.37)
School subjective social status	1.04 (1.00‐1.08)*	1.02 (0.97‐1.06)	1.07 (1.00‐1.14)

* *P* < .05.

** *P* < .01

*** *P* < .001.

**Table 5:** Frequency of physical activity outdoor after school and odds of overweight/obesity^1^ among adolescents

	Total N = 10028	Males N = 4333	Females N = 5695
	OR (95% CI)	OR (95% CI)	OR (95% CI)
Unadjusted			
Frequency of physical activity outdoor			
0 day	1 (reference)	1 (reference)	1 (reference)
1 to 2 days	0.76 (0.63 – 0.91)**	0.73 (0.50 – 1.05)	0.74 (0.57 – 0.96)*
3 to 4 days	0.71 (0.59 – 0.87)**	0.69 (0.53 – 0.91)**	0.66 (0.50 – 0.88)**
All 5 days	0.69 (0.57 – 0.83)***	0.65 (0.47 – 0.89)**	0.59 (0.82 – 0.82)**
Adjusted			
Frequency of physical activity outdoor			
0 day	1 (reference)	1 (reference)	1 (reference)
1 to 2 days	0.79 (0.65 – 0.96)*	0.79 (0.57 – 1.09)	0.80 (0.64 – 1.01)
3 to 4 days	0.76 (0.63 – 0.92)**	0.80 (0.61 – 1.05)	0.76 (0.55 – 1.04)
All 5 days	0.68 (0.55 – 0.84)**	0.74 (0.55 – 1.00)	0.61 (0.43 – 0.86)**
Age	1.01 (0.97 – 1.05)	1.01 (0.95 – 1.07)	1.00 (0.95 – 1.06)
Sex			
Female	1 (reference)		
Male	1.42 (1.14‐1.76)**		
Ethnic background			
White	1 (reference)	1 (reference)	1 (reference)
Black	1.66 (1.36‐2.04)***	1.06 (0.58‐1.93)	2.60 (1.62‐4.15)***
East/Southeast Asian	0.70 (0.56‐0.88)**	0.88 (0.62‐1.23)	0.52 (0.38‐0.71)***
South Asian	0.77 (0.62‐0.96)*	0.79 (0.59‐1.04)	0.76 (0.57‐1.02)
Other	1.18 (0.96‐1.44)	1.09 (0.80‐1.48)	1.29 (1.01‐1.64)*
Subjective socioeconomic status	0.92 (0.88‐0.97)**	0.89 (0.83‐0.94)***	0.98 (0.91‐1.04)
Father education level	0.96 (0.93‐0.99)**	0.96 (0.91‐1.01)	0.95 (0.90‐1.00)*
Mother education level	1.00 (0.96‐1.05)	1.00 (0.93‐1.07)	1.02 (0.96‐1.08)
Parental support	1.09 (1.02‐1.16)*	1.18 (1.07‐1.29)**	0.99 (0.93‐1.06)
School subjective social status	0.94 (0.90‐0.99)**	0.94 (0.88‐1.00)	0.94 (0.90‐3.19)*

* *P* < .05.

** *P* < .01.

*** *P* < .001.

1 World Health Organization definition of overweight/obesity.

### 3.1 Overall sample analysis

The association was more robust for compliance with the physical activity recommendation, with adjusted ORs ranging from 1.90 (95% CI, 1.43‐2.51) for 1 to 2 days to 31.85 (95% CI, 23.40‐43.35) for all 5 days of physical activity outdoor after school (Table 2). One day or more (ie, from 1‐2 d to all 5 d) of physical activity outdoor was significantly associated with greater odds of compliance with the physical activity recommendation (Table 2) and lower odds of overweight/obesity (Table 5). Whereas, 3 days or more (ie 3‐4 d and all 5 d) of physical activity outdoor after school was associated with greater odds of compliance with the screen time recommendation (Table 3). As shown in Table 4, univariate analyses showed that one or more days of physical activity outdoor was significantly associated with greater odds of compliance with the sleep recommendation. However, results were no longer significant after adjusting for covariates.

### 3.2 Sex‐specific analyses

In both male and female adolescents, sex‐specific analyses showed the following results. First, 1 day or more of physical activity outdoor was significantly associated with greater odds of compliance with the physical activity recommendation (Table 2). Second, 3 days or more of physical activity outdoor after school was associated with greater odds of compliance with the screen time recommendation in both male and female adolescents (Table 3). Finally, engaging in physical activity outdoor after school on all 5 days was associated with greater odds of compliance with the sleep duration (Table 4) and with lower overweight/obesity prevalence (Table 5).

Significant gender differences were observed in the associations of outdoor physical activity after school with compliance with the physical activity (Table 2) and the sleep duration (Table 4) recommendations. After adjusting for covariates, females who performed outdoor physical activity after school on 1 to 2 days had greater odds of compliance with the sleep duration recommendation compared with their male counterparts (Table 2). With respect to compliance with the sleep duration recommendation, outdoor physical activity after school on all 5 days was associated with greater odds of compliance with the sleep duration recommendation among males (OR = 1.53; 95% CI, 1.01‐2.31), but not females (OR = 0.92; 95% CI, 0.65‐1.30) (Table 4). No sex differences were observed in the association between the number of days spent physically active outdoor and adherence to the screen time recommendation.

## 4 DISCUSSION

This study investigated the relationships between frequency of outdoor physical activity after school, compliance with the physical activity, screen time, and sleep duration recommendations, and excess weight among adolescents. Collectively, there was a positive gradient between the number of weekdays spent physically active outdoor after school and compliance with the physical activity and screen time recommendations, while a negative gradient with overweight/obesity was observed. We also found that outdoor physical activity after school on all 5 days was associated with greater odds of compliance with the sleep duration recommendation among males, but not females. These findings support the hypothesis that more frequent outdoor physical activity after school is associated with better compliance with the physical activity, screen time, and sleep duration recommendations and healthy weight among adolescents.

The present findings are in line with previous studies that have reported a significant relationship between outdoor time and healthy active living indicators.^7^ For example, Schaefer et al^10^ found that spending more time outdoors after school was associated with more MVPA, better compliance with the physical activity recommendation, less time in sedentary activities, and higher cardiorespiratory fitness in a sample of 306 Canadian youth aged 9 to 17 years old.^10^ Similarly, Larouche et al^8^ in another Canadian study of 1,159 children aged 7 to 14 years has recently found that children reporting more time outdoors were more active, less sedentary, and less likely to have relationship problems with their peer, relative to those who spent less time outdoors.^8^ However, previous studies have mainly focused on time spent outdoor. The present study extends previous research by capturing a specific type of activity youth engage in while outside (ie, outdoor physical activity) and the frequency of such an activity. Results showed that regular physical activity outdoor after school is associated with better compliance with the physical activity, screen time, and sleep duration recommendations (males only) and lower overweight/obesity prevalence among adolescents.

Among the three components of the 24‐hour movement guidelines examined in the present study, frequency of outdoor physical activity was most strongly associated with compliance with the physical activity recommendation. These findings, along with the positive gradient observed between outdoor physical activity and compliance with the physical activity recommendation, provide further support to the affirmation that unstructured outdoor activities after school is a good way to increase children's physical activity.^33^ With only 23.3% of the respondents in this sample meeting, the current physical activity guidelines of at least 60 minutes of MVPA per day, there is a need for heightened efforts to increase this prevalence. Our findings suggest that outdoor physical activity after school should be encouraged to increase compliance with the physical activity recommendation among adolescents.

In contrast to the findings from the current study indicating that frequency of outdoor physical activity after school is associated with greater odds of compliance with the screen time recommendations, LeBlanc et al^34^ found that greater outdoor time was associated with a higher screen time score in a sample of over 5000 children from 12 different countries who participated in the International Study of Childhood Obesity, Lifestyle and the Environment (ISCOLE). The discrepant findings are likely because of the methodological differences, including the age of study participants and the measure used. ISCOLE was conducted among children aged 9 to 11 years. However, the current study encompassed youth aged 11 to 20, and older students were oversampled. The use of different measures for the independent variable (frequency of outdoor physical activity after school vs. outdoor time before school, after school, and on weekends) could also explain the observed differences. School‐aged children and adolescents may have the opportunity to have more screen time during the weekends than on weekdays.^35^, ^36^ Therefore, future research is desired to examine the behaviours during the week and separately on the weekends and to determine whether regular physical activity outdoor could result in compliance with the screen time recommendation among adolescents.

Research studies examining the relationship between outdoor physical activity and sleep duration among adolescents are very sparse. Our finding that outdoor physical activity is not associated with compliance with the sleep duration recommendation in the total sample is consistent with a recent study by Lin et al.^14^ who found that outdoor active play was not associated with sleep duration, chronology, and efficiency in a sample of 433 children aged 10–13 years. Other studies have also found that physical activity is not associated with sleep duration among younger children.^37^, ^38^, ^39^ However, the current study extends these findings by observing an important sex difference in this sample of adolescents. Results showed that outdoor physical activity after school on all 5 days was associated with greater odds of compliance with the sleep duration recommendation among males, but not females. This could be explained by the fact that males are more active in general and they spend more time outside than girls^21^ so they may need to sleep more to recover. Outdoor physical activities among females may not be sufficiently intense to have benefits on sleep duration. Regardless, our results provide evidence of possible benefits of outdoor physical activity among adolescents. Future research is needed to replicate and further understand the sex difference between outdoor physical activities and sleep duration among adolescents.

Strengths of this study include the use of a large and representative province‐wide sample of adolescents, the examination of sex‐specific relationships, and the adjustment for important covariates. However, an important limitation of our analysis is that it was based on cross‐sectional data, which precludes causal inferences. Future studies should utilize longitudinal data to better examine directionality and temporality in the findings. In addition, responses were self‐reported, which may have caused some recall and social desirability bias. Future studies should rely on objective measures of physical activity, sedentary behaviour, sleep duration, and adiposity. Another limitation is related to the fact that the survey assessed only weekday outdoor physical activity.

In conclusion, our results show that regular physical activity outdoor after school is associated with better compliance with the physical activity, screen time, and sleep duration recommendations (males only) and lower overweight/obesity prevalence among adolescents. These findings support previous observations and suggest that outdoor physical activity after school could be a good behavioural target to increase compliance with the physical activity and screen time recommendations and possibly tackle excess weight among adolescents. However, future research using longitudinal design is needed to confirm temporality and clarify the observed sex differences.

## FUNDING

The Ontario Student Drug Use and Health Survey, a Centre for Addiction and Mental Health initiative, was funded in part through ongoing support from the Ontario Ministry of Health and Long‐Term Care, as well as targeted funding from several provincial agencies. The funders had no involvement in study design, collection, analysis, and interpretation of data; writing the report; or the decision to submit the report for publication.

## CONFLICT OF INTEREST STATEMENT

All authors declare no conflict of interest.
